# Innovative Hemp Shive-Based Bio-Composites: Part I: Modification of Potato Starch Binder by Sodium Meta-Silicate and Glycerol

**DOI:** 10.3390/ma17194911

**Published:** 2024-10-08

**Authors:** Laura Vitola, Ina Pundiene, Jolanta Pranckeviciene, Diana Bajare

**Affiliations:** 1 Institute of Sustainable Building Materials and Engineering Systems, Riga Technical University, Kipsalas 6A, LV–1048 Riga, Latvia; 2 Laboratory of Concrete Technologies, Institute of Building Materials, Vilnius Gediminas Technical University, Sauletekio av. 11, LT–10223 Vilnius, Lithuaniaina.pundiene@vilniustech.lt (I.P.)

**Keywords:** potato starch binder, hemp shives, bio-composite, sustainable building materials, sodium metasilicate, glycerol

## Abstract

The growing demand for sustainable building materials has boosted research on plant-based composite materials, including hemp shives bound with biodegradable binders. This study investigates the enhancement of potato-starch-based binders with sodium metasilicate and glycerol to produce eco-friendly bio-composites incorporating hemp shives. Potato starch, while renewable, often results in suboptimal mechanical properties and durability in its unmodified form. The addition of sodium metasilicate is known to improve the mechanical strength and thermal stability of starch-based materials, while glycerol acts as a plasticizer, potentially enhancing flexibility and workability. Bio-composites were produced with varying concentrations of sodium metasilicate (0–107% by mass of starch) and glycerol (0–133% by mass of starch), and their properties were evaluated through thermal analysis, density measurements, water absorption tests, compressive strength assessments, and thermal conductivity evaluations. The results demonstrate that sodium metasilicate significantly increases the bulk density, water resistance, and compressive strength of the bio-composites, with enhancements up to 19.3% in density and up to 2.3 times in compressive strength. Glycerol further improves flexibility and workability, though excessive amounts can reduce compressive strength. The combination of sodium metasilicate and glycerol provides optimal performance, achieving the best results with an 80% sodium metasilicate and 33% glycerol mixture by weight of starch. These modified bio-composites offer promising alternatives t2 o conventional building materials with improved mechanical properties and environmental benefits, making them suitable for sustainable construction applications.

## 1. Introduction

In recent years, there has been growing interest in the development of sustainable and environmentally friendly building materials due to political restrictions [1].

The need for sustainable building materials has driven the exploration of plant-based raw materials and biodegradable binders. Hemp shives, a byproduct of hemp processing, offer a lightweight and renewable reinforcement material for bio-composites [2,3]. When combined with a modified potato starch binder, hemp shives can contribute to the development of eco-friendly and high-performance building materials [2].

The results of the life cycle analysis of plant-based bio-composites indicate that these materials have a lower environmental impact compared to other conventional building materials. In some cases, they have better mechanical performance, hence improved energy efficiency and lower emissions [4,5]. Improving the behavior of bio-composites by adding different additives to their composition—for example, to improve fire resistance and/or bio-resistance—makes the materials less environmentally friendly [6].

A wide variety of plant-based aggregates are used in the production of bio-composites (e.g., hemp and flax shives, oat and wheat husks, etc.) [2,7]. Using hemp shives over other types of plant-based aggregates comes with several justifications based on various factors including availability, sustainability, strength, versatility, and ecological and health benefits. Hemp plants grow quickly, they provide soil health by preventing erosion and their cultivation contributes to less water and pesticides compared to cotton or other plants used as aggregates for material production. Very important factors for the use of hemp shives is their very low price and easy production process without chemical treatment [8]. Plant-derived fillers have different physical properties and chemical compositions and therefore also influence the properties of bio-composites [9,10,11]. For example, hemp bio-composites exhibit significant sound absorption, i.e., up to 90% in the frequency range from 0 to 500 Hz [12], when used in a multi-layer formulation. These materials have the potential to decrease the environmental impact of the building sector while maintaining or improving the performance characteristics needed for various applications. One of the critical components in the development of bio-composites is the choice of binder. Bio-composites, which utilize natural fibers or shives and bio-degradable binders, have emerged as a promising alternative to traditional construction materials [13,14,15]. Potato starch, a renewable and biodegradable material, has been widely studied as a binder due to its availability and cost-effectiveness [16]. However, its use in its unmodified form often results in bio-composites with suboptimal mechanical properties and durability. To address these limitations, researchers have explored the modification of potato starch binders with various additives to enhance their performance [17,18,19].

The addition of sodium metasilicate and glycerol to potato starch binders is a promising method for creating bio-composites with increased density, water resistance and mechanical strength. These improvements have the potential to expand the use of bio-composites in sustainable construction, providing an environmentally friendly alternative to traditional materials. Further research is recommended to improve binder formulations for targeted applications, optimizing both performance and environmental sustainability.

This study focuses on the modification of potato starch binders with sodium metasilicate solution and glycerol to produce bio-composites with hemp shives. Sodium metasilicate is known for its ability to improve the mechanical properties and durability of starch-based materials [20], while glycerol acts as a plasticizer, enhancing the flexibility and workability of the binder [21]. The primary objectives of this research were to evaluate the effects of these modifications on the bulk density, water resistance, and compressive strength of the resulting bio-composites.

## 2. Materials and Methods

### 2.1. Raw Materials

As the main compound for binders, commercially available potato starch (Ltd. Aloja Starkelsen, Municipality of Aloja, Latvia) was used. The bulk density of used starch was 595 kg/m^3^, amylose content was 26.9%, and moisture content was 15.1%, but gelatinization temperature was 64 °C.

The sodium metasilicate solution (Na_2_SiO_3_ ‧ xH_2_O) (Ltd. Vincents Polyline, Municipality of Carnikava, Latvia) was used as one of the additives to modify the binder. The density of the used solution was 1.37 g/cm^3^, the molar SiO_2_:Na_2_O ratio was 3.22, and the boiling point was 100 °C.

As a second additive for the modification of binders, commercially available glycerol (C_3_H_8_O_3_) was used (Ltd. Farmacom, Kharkiv, Ukraine). The relative density of used glycerol was 1.260 g/cm^3^, the refractive index was 1.47, the melting point was 17.9 °C and the boiling point was 290 °C. It can absorb up to 40% of its own weight in water from the atmosphere. 

For the preparation of bio-composite samples, filler hemp shive aggregates (UPB Naturalus pluostas, Kedainiai, Lithuania) resulting from a hemp fiber separation process were used [3]. Hemp shives consist of shive particles up to 40 mm in length, fibers (2.49% by mass), a bulk density of 82 kg/m^3^, 335% water absorption after 24 h immersion, and 4.92% humidity content at room temperature, but thermal conductivity of 0.043 W/(m·K).

### 2.2. Mixture Design and Sample Preparation

In this study, both potato-starch-based binders and bio-composites with hemp shives were prepared and studied. Binders were produced in order to be able to analyze more precisely the effect of sodium metasilicate solution and glycerol on the properties of the binder and the resulting bio-composite by excluding the effect of hemp shives. The compositions of studied binders are presented in Table 1, but the studied bio-composites were prepared in accordance with Table 2.

**Figure 1:**
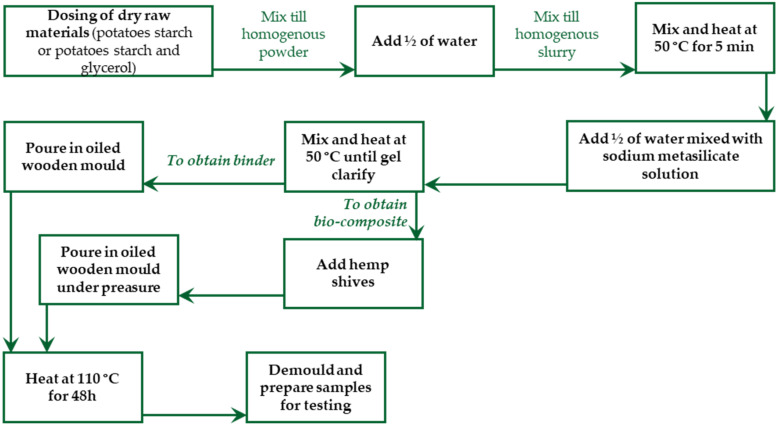
Scheme of binder and bio-composite preparation process.

The preparation procedure scheme of the studied binders and bio-composites after demoulding the samples is given in Figure 1.

### 2.3. Testing Methods

The thermal evolution of the binders was studied by thermogravimetric analysis (TGA) and differential scanning calorimetry (DSC). Tests were carried out using METTLER TOLEDO TGA/DSC 3+, STARe System thermobalance (Mettler Toledo, Columbus, Ohio, the US.). Samples were tested up to 6000 °C in an atmosphere of 10 °C/min. A Varian FTS 800 FT-IR Scimitar Series spectrometer (Varian, Palo Alto, CA, USA) was used to obtain FTIR spectra in a range from 2000 to 400 cm^−1^ with a resolution of 4 cm^−1^. For the FTIR measurements, 1 mg of each sample was mixed with 300 mg of KBr separately. The scanning electron microscopy (SEM) of both binder and bio-composite microstructures was performed using Mira/LMU (Tescan, Brno, Czech Republic). The bulk density of bio-composites was determined according to EN 1602 [22] for specimens, where the size was the same as for mechanical property testing. The capillary water absorption of bio-composites was carried out according to the requirements of EN 1609 [23] (method A) for 24 h. Compressive stress of bio-composites at 10% and 20% of deformation was tested according to the EN 826 method [24] for cubic samples (50 × 50 × 50 mm) using a computerized machine H10KS (Hounsfield, Surrey, UK) with a maximum loading force of 10 kN, a loading accuracy of ±0.5% and a loading speed accuracy of ±0.05%. The thermal conductivity of bio-composites was determined in accordance with EN 12667 [25] for prismatic samples (250 × 250 × 50 mm). The test was carried out using a LaserComp FOX 304 (LaserComp, New Castle, Delaware, the USA) computerized testing machine, which has measurement limits from 0.01 to 0.50 W/(m·K) and a measuring accuracy of ~1%. The difference between measuring plates was 20 °C and the average test temperature was 10 °C.

## 3. Results

### 3.1. Modification of Starch Binder

The properties of five selected binders (Table 1) were determined in order to characterize the influence of the binder composition (i.e., sodium metasilicate solution and glycerol) on the material properties, excluding the influence of the aggregates (hemp shives).

**Figure 2:**
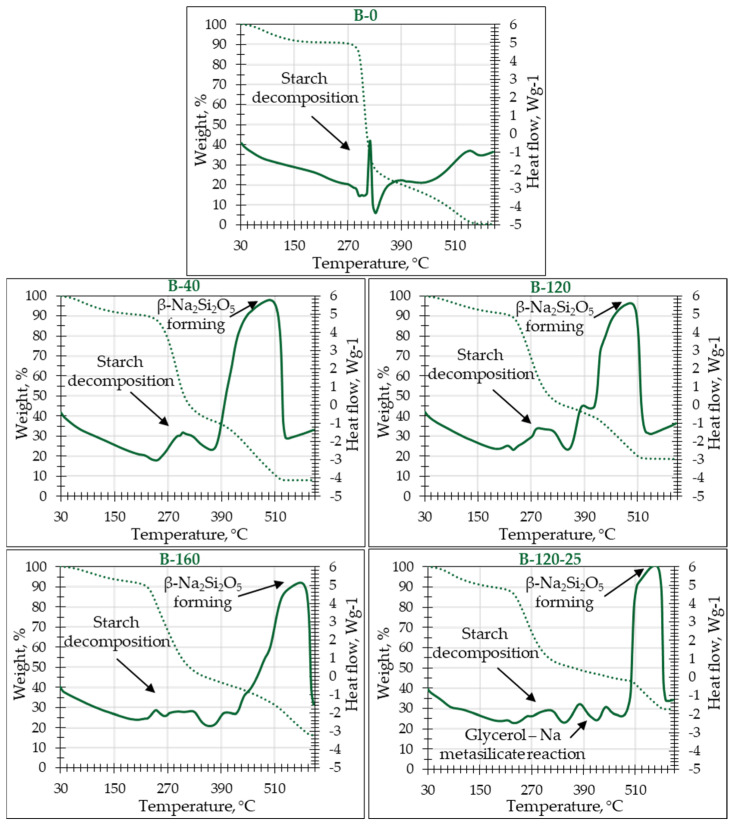
Differential scanning calorimetry (DSC) (solid line) and thermogravimetric analysis (TGA) (dotted line) of studied potato starch binders.

In order to indicate the thermal stability and the decomposition of the binder at elevated temperatures, the TGA/DSC test was carried out (Figure 2). The behavior of potato starch at a certain temperature was mainly determined by the amount of polymer compounds and therefore also by the crystalline and amorphous region. According to the literature, potato starch is characterized by relatively less degradation at elevated temperatures, but this starts at lower temperatures [26].

Figure 2 illustrates the DSC curves, where the maximum peaks correspond to the points of maximum mass loss rate. The DSC curve for sample B-0 distinctly differs from the other samples, likely due to its unique composition, as indicated in Table 1. Notably, sample B-0 contains a higher proportion of polymer compounds, which influences its thermal behavior.

The DSC curves were used to study the thermal transformations occurring during the heating process. In the first stage, water evaporation was observed across all samples within the temperature range of 120 °C to 150 °C, with sample B-0 exhibiting a mass loss of 10%. Post-evaporation, the mass remained stable up to 280 °C.

For sample B-40, water evaporation concluded at 160–175 °C with an 8.5% mass loss, followed by stable mass retention up to 230 °C. Sample B-120 exhibited similar behavior, with water evaporation ceasing at 160–180 °C with a 7% mass loss, and then remaining stable until 220 °C. In sample B-160, water evaporation also occurred within 160–180 °C, with a 5% mass loss, stabilizing up to 220 °C. Sample B-120-25, which includes glycerol, showed water evaporation ending at 150–160 °C with an 11% mass loss, and stability until 250 °C.

A rapid decomposition of starch in sample B-0 occurs between 290 and 330 °C, resulting in a 75% weight loss. Further thermolysis of starch in the 390–520 °C range leads to an additional 25% weight loss.

For samples containing sodium metasilicate, the TGA and DSC curves display two distinct regions of weight loss, reflected by two peaks in the DCS curves. This suggests a two-step decomposition of the starch silication products, with a characteristic peak of starch decomposition between 250 and 370 °C, followed by another peak associated with deep thermolysis of starch between 390 and 600 °C. In sample B-120-25, the decomposition of glycerol is observed within the 200–260 °C range [27], alongside an additional exothermic effect between 420 and 440 °C, where glycerol decomposition products react with sodium metasilicate.

In samples containing sodium metasilicate, the temperature range of 300–400 °C shows a polymerization of sodium metasilicate, accompanied by some mass loss. Initially, amorphous sodium metasilicate begins to polymerize, forming β-Na_2_Si_2_O_5_ crystals around 400 °C and crystallizing into SiO_2_ modification cristobalite at approximately 600 °C. These phases coexist up to 700 °C [28].

Mass losses during starch decomposition for samples B-40, B-120, B-160, and B-120-25 were 55%, 50%, 48%, and 45%, respectively. It is evident that increasing the amount of sodium metasilicate raises the thermolysis temperature of starch, indicating positive interactions among the components. A higher decomposition temperature is advantageous for practical applications, such as improved fire resistance.

During the polymerization of sodium metasilicate, mass losses for B-40, B-120, B-160, and B-120-25 samples reached 64%, 60%, 58%, and 50%, respectively. Starch thermolysis resulted in mass losses of 91%, 84%, 81%, and 70% for the respective samples. As the amount of sodium metasilicate increases, the thermolysis temperature of starch increases, while the total mass loss decreases. The addition of glycerol exerts the most significant influence on thermolysis temperature, with sample B-120-25 exhibiting the highest onset temperature for the process. Furthermore, B-120-25 demonstrated the lowest overall mass loss, suggesting that sodium metasilicate, particularly when combined with glycerol, mitigates complete starch thermolysis. This is evident from the residual weight results, where sample B-0 exhibited complete weight loss, whereas samples containing sodium metasilicate retained 9–19% of their weight, and the sample with added glycerol (B-120-25) retained up to 30%.

**Figure 3:**
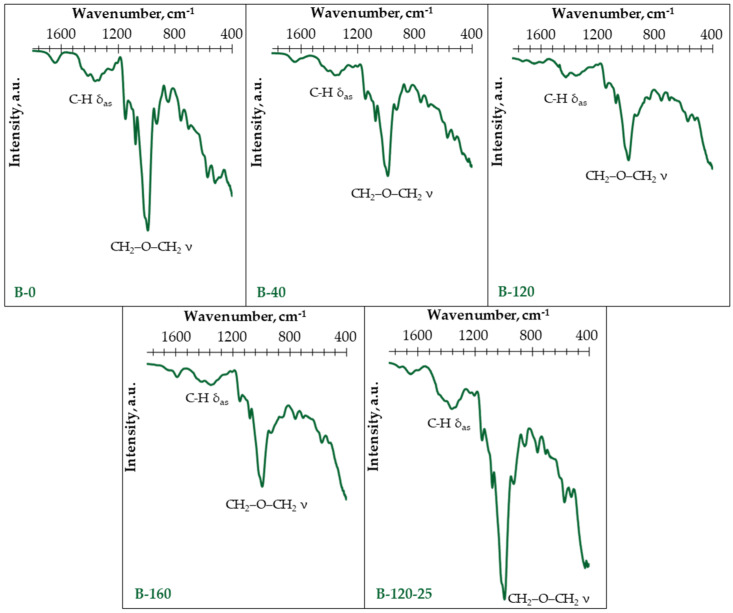
FTIR transmittance spectra of studied potato starch binders.

To characterize the structural changes in potato starch binders due to modification in binder composition, FTIR spectral curves (Figure 3) were made for the binders given in Table 1.

Regarding samples B-120, B-160 and B-120-25, relatively small peaks appear at 1695 cm^–1^, which can be attributed to the bending of molecular coordinated water within the SiO_2_ structure [29,30]. While the band appearing at 1637 cm^−1^ (for sample B-120-25) corresponds to the glycerol present in the sample, i.e., representing the C=O band of an amidic group (N–C=O) [31]. As the samples B-0 and B-40 have less contaminants in the composition (Table 1), in their spectra, peaks at 1630 cm^−1^ appear, which are characteristic of water absorbed in the amorphous regions of starch [32].

The bands at 1370 cm^−1^ correspond to the C-H symmetric bending in potato starch [33]. Both bands at 1060 and 1010 cm^−1^ correspond to the stretching vibration of CH_2_–O–CH_2_, but the band at 835 cm^−1^ corresponds to the CH_2_–O–CH_2_ ring mode stretching vibration [34].

The spectrums of all samples present three bands at 1163, 1060, and 947 cm^−1^, that are, respectively, the characteristics of P=O, C–O–P, and O–P–O of the phosphate group in potato starch [35].

**Figure 4:**
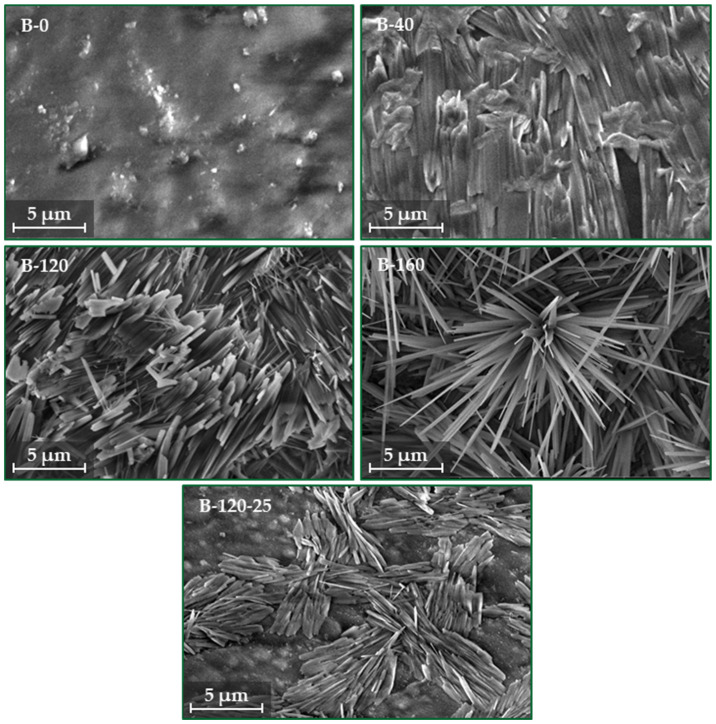
Microstructure of obtained potato starch binder.

The microstructure of the studied binders is given in Figure 4. B-0 presents a mostly smooth and homogeneous surface with very few crystalline structures, which might be a sign that the starch is not properly gelatinized. Meanwhile, adding the sodium metasilicate to the composition significantly increases the crystalline structures compared to reference sample B-0. The addition of sodium metasilicate starts the activation process, leading to the formation of some crystalline phases. The crystalline structure becomes more pronounced, with a denser and more organized needle-like morphology. The higher amount of sodium metasilicate enhances the polymerization process, resulting in a more extensive crystalline formation. Sample B-160 presents the most extensive needle-like crystalline structures, indicating a highly activated matrix. The increased amount of sodium metasilicate leads to further enhanced crystallization and polymerization of the starch. As can be seen in the images, B-120-25 has a similar needle-like crystalline structure to B-120 but with some differences in morphology. The addition of glycerol likely impacts the crystallization process, potentially acting as a plasticizer, which could explain a slightly different organization or distribution of the crystals.

The composition of the binders impacts the microstructure, which is crucial for ensuring the material properties such as mechanical strength, durability, and application potential of both binder and bio-composites.

### 3.2. Bio-Composites Bounded by Potato-Starch-Based Binder

**Figure 5:**
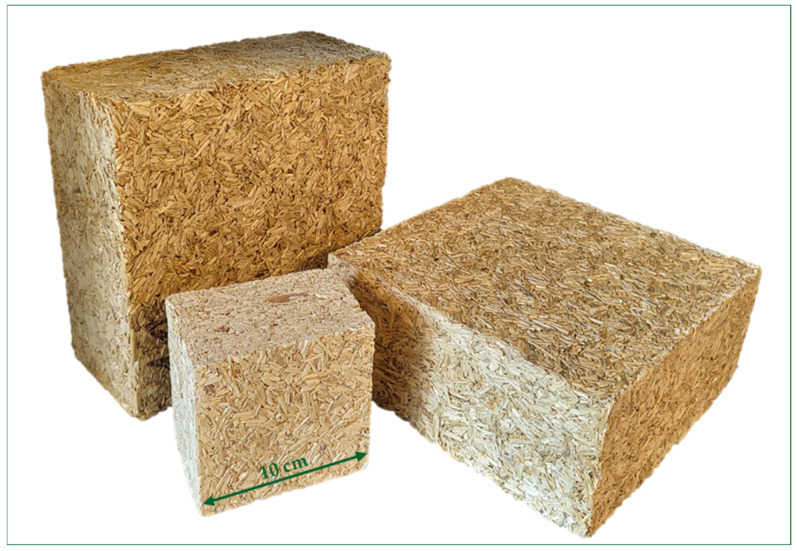
The obtained bio-composites bounded by potato starch binder.

In this study, samples of fifteen different compositions (Table 2) (i.e., one reference composition with pure potato starch, eight compositions with no potato starch modified by sodium metasilicate solution, and six compositions with potato starch modified by sodium metasilicate solution and glycerol) were studied. The obtained material can be seen in Figure 5; it has a stable structure and is easy to process (i.e., cut).

**Figure 6:**
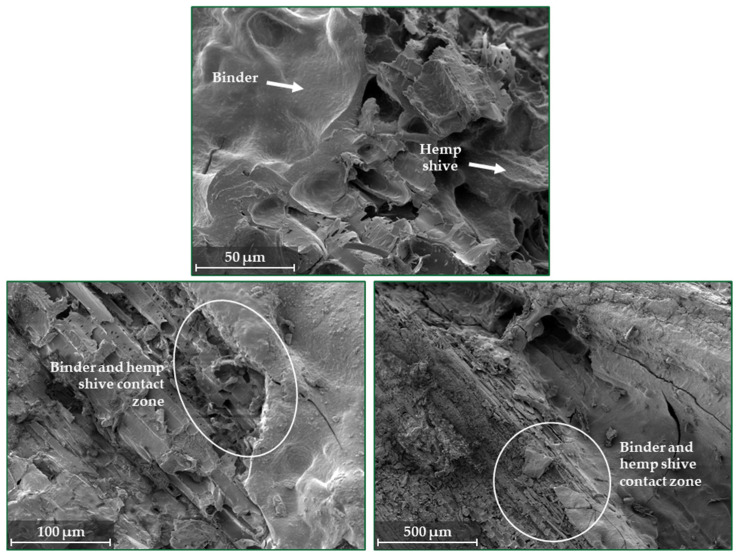
The microstructure of obtained bio-composites bounded by a potato starch binder modified by sodium metasilicate solution and glycerol (BC-120-25).

In Figure 6, the contact zone between a hemp shive and a binder in different magnifications is shown. The different magnifications provide a comprehensive view, illustrating a cohesive and potentially relatively high-performance lightweight material. A well-integrated structure of bio-composite where the binder effectively surrounds and penetrates the hemp shive can be seen in the given SEM images. The good dispersion and adhesion of the binder to the hemp shive indicate that the composition of the B-120-25 binder is optimal to obtain a durable bio-composite material. Meanwhile, the presence of glycerol in the binder composition might contribute to the flexibility and workability of the composite, aiding in the good distribution and adhesion observed in the SEM images.

In this study, bio-composites with different compositions (Table 2) were prepared. The reference bio-composite sample (BC-0) presents the bulk density of 197 kg/m^3^, the capillary water absorption after 24 h immersion of 22.9 kg/m^2^, compressive strength of 0.7 MPa at 10% deformation and 1.0 MPa at 20% deformation.

**Figure 7:**
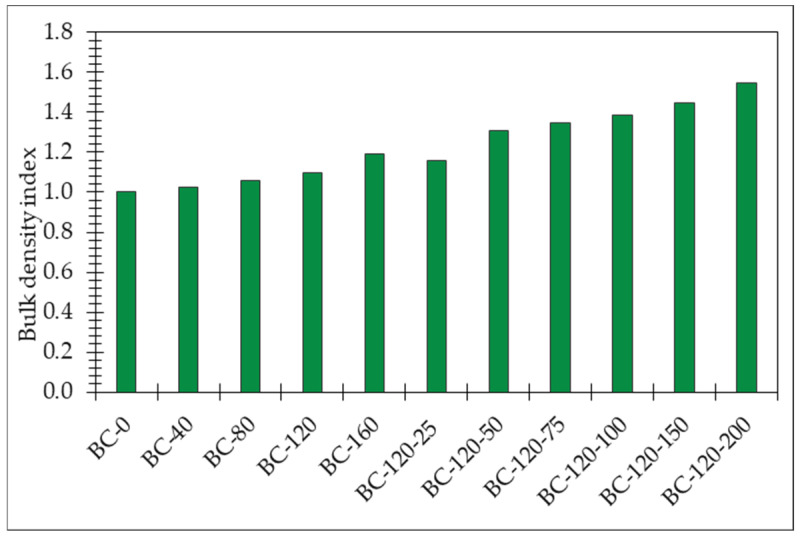
The index of the bulk density of obtained bio-composites bounded by potato starch binder modified by sodium metasilicate solution and glycerol.

The bulk density index of obtained bio-composites is presented in Figure 7. As can be seen in the graph, modification of potato starch binders ensures the increase in bulk density of the resulting bio-composites. Adding 40 g of the sodium metasilicate solution to 160 g on each 150 g of potato starch (i.e., 27–107% of potato starch mass) to the composition of binder (Table 2) causes a bulk density increase of 3–19% in comparison to BC-0. Similar to the addition of sodium metasilicate, the addition of glycerol to the binder causes an increase in the density of the resulting bio-composite material. Potato starch binder modification by sodium metasilicate ensures the increase in the bulk density of the resulting bio-composite from 16% to 55% in comparison to the reference samples (bio-composites obtained with unmodified potato starch binder). The glycerol by itself increases the bulk density of bio-composites from 5% to 41% in comparison to the same composition with no additional glycerol (i.e., BC-120).

**Figure 8:**
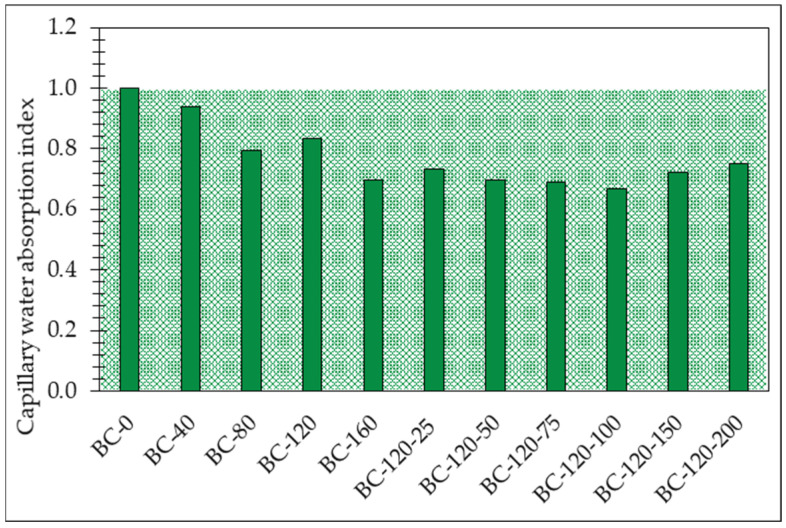
The index of the capillary water absorption of obtained bio-composites bounded by potato starch binder modified by sodium metasilicate solution and glycerol. (green background: acceptable results).

Modification of potato starch binder by sodium metasilicate solution and glycerol affects the structure of the resulting material, which is also reflected in the capillary water absorption results in Figure 8. Adding sodium metasilicate solution to potato starch binder decreases the bio-composite capillary water absorption from 6.1% to 33.2%. Although glycerol also contributes to a reduction in water absorption, its impact is less significant compared to sodium metasilicate. This suggests that sodium metasilicate effectively enhances the moisture resistance of the bio-composites, making them more suitable for applications where water exposure is a concern.

**Figure 9:**
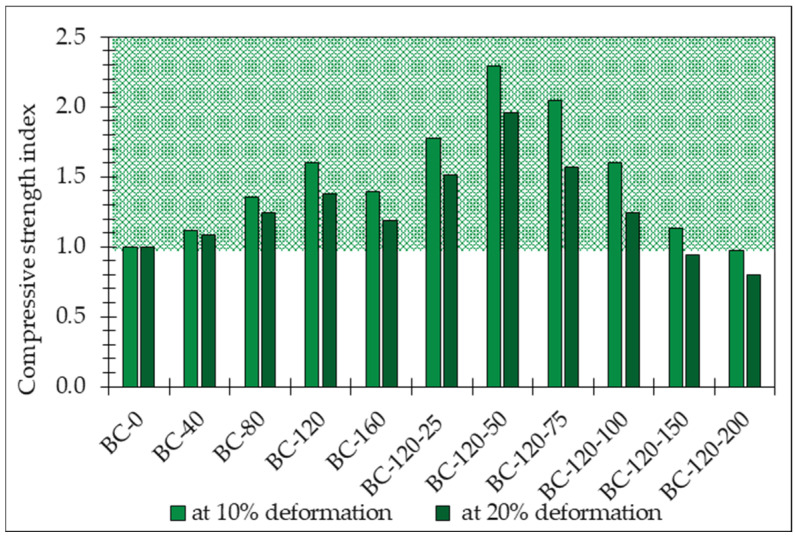
The index of compressive strength of obtained bio-composites bounded by potato starch binder modified by sodium metasilicate solution and glycerol.

The compressive strength behavior of the bio-composites depending on the composition (Table 2) is illustrated in Figure 9. The results reveal a notable increase in compressive strength with the addition of sodium metasilicate, i.e., from 11.5 to 59.8% at 10% deformation and from 8.7 to 37.9% at 20% deformation. Meanwhile, sodium silicate and glycerol additive increase the resulting bio-composite compressive strength depending on the amount of added glycerol. Samples BC-120-50 demonstrate the optimal composition of the obtained bio-composites from the point of compressive strength.

The thermal conductivity was tested for six samples in purpose to establish the impact of the sodium metasilicate and both sodium metasilicate and glycerol on the thermal properties of the samples: reference sample B-0 without sodium metasilicate and samples B-40, B-80, B-120, B-160 and B-120-25 with the addition of glycerol. For the studied samples, thermal conductivity was in the range of 0.062 to 0.068 W/(m·K).

## 4. Discussion

This study shows that modification of potato starch binder with sodium metasilicate solution and glycerol significantly improves the properties of the resulting bio-composites.

The increase in the water evaporation interval in samples with sodium metasilicate is related to the loss of water from sodium metasilicate. It is known that sodium metasilicate degrades at a temperature interval of 150–300 °C [36]. Sodium metasilicate undergoes gradual dehydration, losing two water molecules at approximately 170–175 °C [37]. This effect slightly delays water evaporation in these samples. Additionally, glycerol vaporization begins at 120 °C.

The incorporation of sodium metasilicate significantly increases the bulk density of the bio-composites, with improvements ranging from 3% to 19% over the unmodified sample (BC-0). This increase is more pronounced compared to the effect of glycerol alone, which enhances density by 5% to 41% relative to the same binder without glycerol. Sodium metasilicate’s impact on density can be attributed to its role in polymerizing and reinforcing the starch matrix, leading to a denser material structure. Compared to the structure formed in the sample just with sodium metasilicate, a lower amount of heat exchange which takes place through the air occurs, compared to the sample with an addition of glycerol. Compared to the BC-120 sample, the addition of glycerol increases density, and in the denser sample structure, heat transfer occurs through the solid frame and the low alteration in heat transfer that takes place through the air when the density of the composite increases [38].

In terms of moisture resistance, sodium metasilicate exhibits a more substantial effect, reducing capillary water absorption by 6.1% to 33.2% compared to the unmodified binder. Glycerol also contributes to a reduction in water absorption but to a lesser extent. This finding indicates that sodium metasilicate enhances the water resistance of the bio-composites, making them more suitable for applications where exposure to moisture is a concern. The greater amount of liquid phase, which can partially fill up the voids, enables improved interaction between the hemp shives, starch and sodium metasilicate and glycerol [39].

The mechanical properties of the bio-composites are notably improved with the addition of both sodium metasilicate and glycerol. The compressive strength of the bio-composites increases significantly, with sodium metasilicate contributing to an increase of 11.5% to 59.8% at 10% deformation and 8.7% to 37.9% at 20% deformation. The addition of glycerol further enhances compressive strength, particularly in compositions with optimal amounts of sodium metasilicate. This improvement in mechanical properties suggests that the modified binders yield bio-composites that are more robust and capable of withstanding load-bearing applications.

Thermal conductivity measurements reveal a moderate increase with the addition of sodium metasilicate, with values ranging from 0.062 to 0.068 W/(m·K). This increase in thermal conductivity correlates with the observed increase in bulk density, as denser materials typically exhibit higher thermal conductivity. The presence of glycerol also influences thermal conductivity, though its effect is less pronounced compared to sodium metasilicate. The modifications in binder composition lead to variations in the thermal properties of the bio-composites, although the differences are relatively minor.

## 5. Conclusions

The modification of potato starch binder by sodium metasilicate and glycerol noteworthily improves the properties of the resulting hemp shive bio-composites. Sodium metasilicate increases bulk density, water resistance, and compressive strength, while glycerol contributes to improved flexibility and workability.

Sodium metasilicate notably increases the bulk density of bio-composites by up to 19.3%, but increases sodium metasilicate solution and glycerol mix by up to 54.8%. The compressive strength of the bio-composites is significantly enhanced with the addition of both sodium metasilicate and glycerol (up to 2.3 times higher), making them suitable for load-bearing applications. At the same time, glycerol should not be added in excessive amounts. When ≥100% glycerol is added by weight of starch, the compressive strength at 20% deformation starts to rapidly decrease compared to the reference.

Sodium metasilicate substantially reduces capillary water absorption, improving the moisture resistance of the bio-composites. This characteristic is crucial for applications where exposure to water is expected, as well as from the point of view of fungal resistance.

The thermal conductivity of the bio-composites increases with the amount of sodium metasilicate, correlating with increased density. The impact of glycerol on thermal conductivity is less pronounced.

According to the results obtained, the most optimal results can be obtained by modifying the potato starch binder by adding sodium metasilicate solution and glycerol (80% and 33% by weight of starch). The observed increases in density, water resistance, and mechanical strength suggest that bio-composites with modified potato starch binders are well suited for sustainable building material applications. The results highlight the potential of these modified binders to enhance the performance of bio-composites, providing a solid foundation for further research aimed at optimizing binder compositions for specific applications.

## Figures and Tables

**Table 1:** Compositions of studied binders.

Composition	Raw Materials for Binder, Mass Parts			
	Potato Starch	Sodium Metasilicate Solution	Glycerol	Water
B-0	150	0	0.0	760
B-40	150	40	0.0	760
B-120	150	120	0.0	760
B-160	150	160	0.0	760
B-120-25	150	120	25	760

**Table 2:** Compositions of studied bio-composites.

Composition	Raw Materials for Binder, Mass Parts				Hemp Shives
	Potato Starch	Sodium Metasilicate Solution	Glycerol	Water	
BC-0	150	0	0	760	500
BC-40	150	40	0	760	500
BC-80	150	80	0	760	500
BC-120	150	120	0	760	500
BC-160	150	160	0	760	500
BC-120-25	150	120	25	760	500
BC-120-50	150	120	50	760	500
BC-120-75	150	120	75	760	500
BC-120-100	150	120	100	760	500
BC-120-150	150	120	150	760	500
BC-120-200	150	120	200	760	500

## Data Availability

The original contributions presented in the study are included in thearticle, further inquiries can be directed to the corresponding author.
